# Net-zero emission targets for major emitting countries consistent with the Paris Agreement

**DOI:** 10.1038/s41467-021-22294-x

**Published:** 2021-04-09

**Authors:** Heleen L. van Soest, Michel G. J. den Elzen, Detlef P. van Vuuren

**Affiliations:** 1 PBL Netherlands Environmental Assessment Agency, The Hague, The Netherlands; 2 Copernicus Institute of Sustainable Development, Utrecht University, Utrecht, The Netherlands

**Keywords:** Climate-change mitigation, Projection and prediction, Energy modelling

## Abstract

Over 100 countries have set or are considering net-zero emissions or neutrality targets. However, most of the information on emissions neutrality (such as timing) is provided for the global level. Here, we look at national-level neutrality-years based on globally cost-effective 1.5 °C and 2 °C scenarios from integrated assessment models. These results indicate that domestic net zero greenhouse gas and CO_2_ emissions in Brazil and the USA are reached a decade earlier than the global average, and in India and Indonesia later than global average. These results depend on choices like the accounting of land-use emissions. The results also show that carbon storage and afforestation capacity, income, share of non-CO_2_ emissions, and transport sector emissions affect the variance in projected phase-out years across countries. We further compare these results to an alternative approach, using equity-based rules to establish target years. These results can inform policymakers on net-zero targets.

## Abstract (web summary)

Over 100 countries have set or are considering net-zero emissions targets. Here, the authors show that a country’s potential for negative emissions and methodological issues affect when countries can reach net-zero, calling for clear internationally agreed definitions and accounting methods.

## Introduction

In the 2015 Paris Climate Agreement^1^, Parties agreed to keep the increase in global average temperature to well below 2 °C above pre-industrial levels and to pursue efforts to limit temperature rise further to 1.5 °C (Article 2). To reach these objectives, Parties further agreed to “reach global peaking of greenhouse gas emissions as soon as possible […] and […] to achieve a balance between anthropogenic emissions by sources and removals by sinks of greenhouse gases in the second half of this century.” (Article 4)^1^. This balance between greenhouse gas (GHG) emission sources and sinks can be defined as GHG emissions neutrality^2^. This is elaborated by Rogelj et al.^3^ who define carbon neutrality as the total annual CO_2_ emissions from all anthropogenic sources being net-zero and GHG emissions neutrality as the sum of all Kyoto GHG emissions being net zero (in CO_2_-equivalent). The latter is also referred to as climate neutrality. The concept of emissions neutrality has gained interest among policy-makers and an increasing number of governments have formulated neutrality targets^4^. The strength of neutrality targets is that they constitute a clear vision for the long-term ambition of climate policy. Earlier, scenarios from integrated assessment models (IAMs) were used to determine neutrality targets at the global level. In most of the cost-optimal scenarios consistent with limiting global warming to 2 °C relative to pre-industrial levels with at least 66% probability, net-zero GHG emissions occurs shortly after 2085; in 1.5 °C scenarios, this occurs between 2060 and 2085, i.e., roughly 25 years earlier^5^. The use of less or no net negative emissions would imply an earlier year of neutrality (phase-out year), achieved through other means such as drastic efficiency improvements. Net-zero CO_2_ emissions occur earlier than net-zero GHG emissions, i.e., between 2065 and 2080 for 2 °C and between 2045 and 2060 for 1.5 °C, on a global level. The exact value of the phase-out year also depends on methodological choices. For instance, the phase-out year depends on the GHG-equivalence metric used (such as the Global Warming Potential, GWP)^6^. It further depends on the interpretation of the word balance in Article 4 of the Paris Agreement^7^, e.g., whether it corresponds to stable global mean temperature, radiative forcing or emissions, and whether it includes only anthropogenic or all GHG sources and sinks^8^.

So far, studies on GHG and carbon neutrality have mostly focused on the global level. However, as more than 100 national governments (e.g., EU, China, Japan and South Africa) and over 800 cities^4^ have set or are considering net-zero emissions targets, it is more policy-relevant to look at the implications at the national level. Therefore, we use a set of scenarios by IAMs that represent major emitting countries individually, to analyse national neutrality targets for major emitting countries (for brevity, we will refer to countries and national, although the EU is not a country). We focus on the phase-out year for CO_2_ and GHG emissions in scenarios consistent with the Paris Agreement temperature targets, the influence of methodological choices and the key factors that could determine the differences between countries. By presenting detailed information for ten countries based on the CD-LINKS database^9^, directly relevant for national policy-making and international negotiations, we go beyond the existing literature. Although IAMs have developed to represent individual countries and current climate policies in more detail, IAMs are not the only tools for analyses such as presented here—national energy system models, e.g., can do so too, often with greater granularity. These tools are already applied jointly to develop national-level pathways that account for national circumstances but still meet the global goals of the Paris Agreement. The results that we present here should be complemented with an assessment of feasible reductions at the national level, considerations of equity and national model results, among others.

## Results

### National phase-out years for large countries

**Fig. 1:**
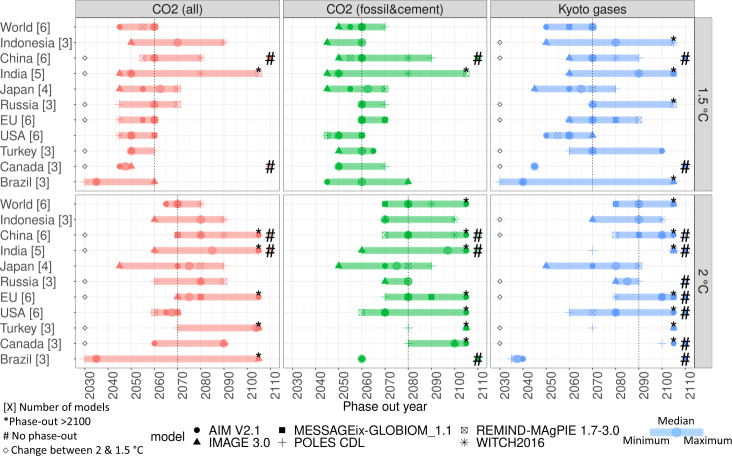
Year when projected emissions reach net zero, per country (number of models representing that country between brackets), for 2 °C and 1.5 °C scenarios, for CO_2_ emissions, CO_2_ emissions from fossil fuels and cement (energy and industrial processes), and total GHG emissions (Kyoto Gases, including land-use emissions). Individual models are indicated by symbols, whereas the bars show the minimum–maximum range (enlarged circles: model median). In some cases, individual models show a phase-out after 2100 in the extrapolated data (indicated by an asterisk) or no phase-out at all (#). Diamonds plotted at the 2030 mark indicate a change between the 2 °C and 1.5 °C scenario in terms of a country reaching net zero earlier than, similar to, or later than global average. Vertical dotted lines indicate the global average phase-out year.

We analysed a set of existing globally cost-optimal scenarios from six IAMs for which detailed, national-level results were available (assuming optimal climate policy to be implemented from 2020 onwards; see “Methods”). The six models included are AIM^10^, IMAGE^11^, MESSAGE-GLOBIOM^12^, POLES^13^, REMIND-MAGPIE^14^ and WITCH^15^ (see also Supplementary Methods). These scenarios can be used to look into cost-optimal phase-out years, without fairness considerations. The scenarios address both 1.5 °C and 2 °C targets (relative to pre-industrial levels, with at least 66% probability of achieving the targets). In the scenario set, global GHG emissions are projected to reach net zero between 2050 and 2070 in 1.5 °C scenarios and after 2080 in 2 °C scenarios. That is consistent with findings in the Special Report on 1.5 °C by the Intergovernmental Panel on Climate Change (IPCC), in which more models and scenarios are included, but for which the required national-level results are not available. CO_2_ is projected to be phased out earlier: between 2045 and 2060 in 1.5 °C scenarios and between 2065 and 2080 in 2 °C scenarios. At the same time, there are clear differences in phase-out years of different countries (Fig. 1). As there are also large differences between the models, we look at both the median and the spread of the model results, and refer the reader to the Supplementary Results for more details.

For the median of the 2 °C scenarios, GHG emissions (including land use) are projected to reach net zero earlier than the global average in Brazil, Japan, Russia (across models) and the United States (with a larger model spread), but later than global average in Canada (across models), as well as in China, EU, India and Turkey (with a larger model spread). Indonesia’s median projected phase-out year is equal to the global average. For most regions, the order is similar in the 1.5 °C scenario, but Canada (now earlier) and Indonesia (now later) are the main exceptions. The difference between Canada and the United States in the 2 °C scenario (only projected by one model) can be explained as follows. That model uses national inventory data for land use, land-use change and forestry (LULUCF) emissions (see next section), unlike the other two models that cover both Canada and the United States. As the inventory data show a sink for the United States but an emissions source for Canada, the United States can phase out emissions earlier than Canada. For CO_2_ only (including land use), countries that reach net-zero emissions earlier than global average are again Brazil and the United States (the former with a large model spread, but it is worth noting that Brazil is only covered by three models, two of which project similar phase-out years). Results are somewhat similar in the 1.5 °C scenario, but now Canada, India and Turkey join the early group. Focusing on fossil CO_2_ only (thus excluding land use), Brazil, Indonesia, Japan and the United States are projected to have net-zero CO_2_ emissions earlier than the global average in the 2 °C scenario (only Canada and the United States in the 1.5 °C scenario). This finding is confirmed by Schaeffer et al.^16^ who show net-zero energy CO_2_ emissions by or before 2050 for Brazil and the United States, based on national model studies. In contrast, Canada, India and Turkey show a later than global average phase-out in the 2 °C scenario (only India and Japan in the 1.5 °C scenario). The other countries have a phase-out year comparable to the global average. Comparing the phase-out years for CO_2_ emissions with those for only fossil CO_2_ shows that countries in which land use is a source of emission (e.g., Indonesia) will see a later phase-out of CO_2_ than of fossil CO_2_ only, whereas in countries in which land-use forms a sink (e.g. Canada), the reverse is true.

All-in-all, this means that Brazil and the United States typically have a phase-out year earlier than the global average, whereas India is projected to reach net-zero emissions later than the global average (in four out of six scenario–source combinations). China and the EU are relatively similar to the global average (namely in four out of six scenario–source combinations and later than global average in the remaining two). The remaining five countries show a mixed picture: results vary across sources of emissions and temperature targets.

Supplementary Table 9 shows additional information on the emissions projections, to support thinking about linking longer-term, net-zero emissions goals to shorter-term action such as formulated in Nationally Determined Contributions (NDCs). For example, GHG emissions are projected to peak in 2020 in many countries that have not yet seen peak emissions and be reduced by between 12% (India) and 36% (Japan, Canada and Indonesia) by 2030 relative to 2015 levels, under the 2 °C scenario. By 2050, these reductions amount to 52% (Brazil) to 72% (USA), and up to 90% (USA) under the 1.5 °C scenario.

### The influence of definitions

**Fig. 2:**
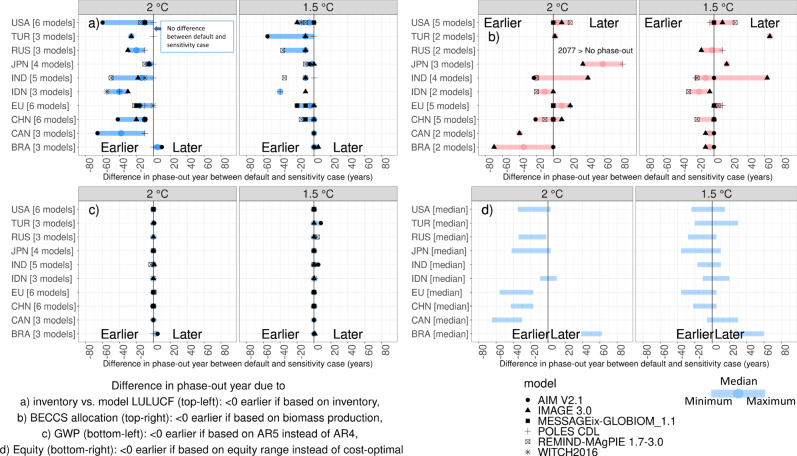
Influence of definitions on projected phase-out years. Change in projected phase-out years for **a** all GHG emissions including land use when harmonizing the model projections towards the countries’ land-use emissions estimates, i.e., by adding the absolute emissions difference in 2010 between the inventory data and the model data to the model projections; values smaller than 0 indicate an earlier phase-out when emissions projections of individual models are harmonized to the inventory LULUCF data. **b** CO_2_ emissions when negative emissions from BECCS are allocated to the biomass producer instead of the carbon-storing country (note that results are shown for fewer models, as POLES did not report the required variable agricultural production of energy crops). **c** The sum of CO_2_, CH_4_, N_2_O and SF_6_ emissions when using 100-year global warming potentials from the Fifth Assessment Report (AR5) of IPCC instead of the fourth (AR4). **d** All GHG emissions when the equity ranges from Robiou du Pont et al.^25^ are used instead of the model median for the default cost-optimal approach, noting that the results reported by Robiou du Pont et al.^25^ do not go beyond 2100, whereas the cost-optimal scenarios do. Therefore, India and Turkey are not shown for the 2 °C scenario, because the equity range included 2100 (which may actually mean somewhere after 2100), while the cost-optimal median phase-out year was calculated as being beyond 2100 in these two cases. Individual models are indicated by symbols, whereas the error bars show the minimum–maximum range from models (enlarged circle: median). Extrapolated emissions data were used to calculate the phase-out year difference, so as to not introduce a bias when calculating differences in phase-out years. Vertical lines at 0 indicate no difference between the default and sensitivity cases. BRA: Brazil, CAN: Canada, CHN: China, EU: European Union (EU27 + UK), IND: India, IDN: Indonesia, JPN: Japan, RUS: Russian Federation, TUR: Turkey, USA: United States.

A number of technical issues has a strong influence on the reported phase-out year at the national level. We explore four that are highly debated but not yet in the context of neutrality targets, i.e., the use of inventory data for LULUCF-related emissions, the allocation of negative emissions, the GWPs and equity considerations (respectively, Fig. 2a–d).

First of all, there are large differences between the land-use change (LUC) emissions produced by the models (and scientific inventories) and LULUCF emissions reported by countries in their national GHG inventories^17–21^. The latter focus on the balance of sinks and sources on managed land, including CO_2_ uptake by forests. On the other hand, the former typically focus on direct human-induced effects of changes in vegetation type. It has been suggested that it is possible to use the inventory data for the base year in combination with the model projections. Figure 2a shows how projected phase-out years change when harmonizing the model projections towards the countries’ reported land-use emission estimates (see also Supplementary Fig. 1 and Supplementary Table 1 of Supplementary Methods and Results). As the inventory data have lower LULUCF emissions mainly due to the sink of the managed forests, net-zero GHG emissions are projected to be reached earlier when using inventory LULUCF data (except for Brazil, see below). In other words, adjusting countries’ GHG and CO_2_ emission projections through harmonization of the LUC CO_2_ emission projections by models with the current (2010) LULUCF emissions from the national inventories data will require countries to phase out GHG emissions earlier. The impacts are quite considerable with the exception of the POLES model^13^, because it uses the inventory data for Annex I countries. In countries where LULUCF emissions play a relatively large role or are uncertain (e.g., Indonesia), the effect is most pronounced. Brazil is a special case, because that is the only country for which the models report lower LUC emissions than the inventory (SEEG^21^), resulting in a later phase-out when using inventory data.

Regarding allocation of negative emissions from bioenergy with carbon capture and storage (BECCS, Fig. 2b), in models these are normally allocated to the country where the carbon is stored. If the allocation of negative emissions from BECCS is changed, ex-post, to the country where the biomass is produced, projected phase-out years change. We have changed the allocation ex-post by using the share in global bioenergy production (see Supplementary Methods and Results) and have calculated the difference in phase-out years as follows: phase-out year of CO_2_ emissions when negative emissions are allocated to the biomass producer (Emissions | CO_2_ | Allocation) − phase-out year of CO_2_ emissions when negative emissions are allocated to the carbon-storing country (default: Emissions | CO_2_*)*. In that case, Brazil, Canada, India (albeit with a large model spread) and Indonesia show earlier net-zero GHG emissions, because these countries produce and export a lot of biomass in the models. On the other hand, the EU, Japan and Turkey show a later phase-out, as these countries generally import biomass. Supplementary Fig. 2 shows emission pathways for two illustrative countries for the default case and the sensitivity cases of LUC data and negative emissions allocation.

The effect of using different GWPs is illustrated by looking at the impact of using 100-year GWP values (excluding feedback^22^) from the IPCC’s Fourth Assessment Report (AR4) and Fifth Assessment Report (AR5), focusing on CO_2_, CH_4_, N_2_O and SF_6_ emissions. We focus on GWP100, as it is prescribed for NDCs, but countries are free to choose an additional metric^23^. We further focus on AR4 and AR5, as GHG reporting and accounting are moving to more recent GWPs, in line with the decisions made at the COP in Katowice. The results in Fig. 2c show that changing the GWPs from AR4 to AR5 does not result in significant shifts in projected phase-out years (up to 8 years earlier or later), similar to findings by Fuglestvedt et al.^7^. Choosing other metrics, such as Global Temperature change Potential^24^, would result in larger effects on phase-out years^6, 7^.

Finally, the effect of equity considerations (Fig. 2d) is also important. As indicated earlier, cost-optimality is only one consideration in target setting. To compare these results to those based on equity principles, we took the most extreme (earliest and latest) phase-out years based on five different equity approaches as presented by Robiou du Pont et al.^25^ (see their Supplementary Tables S3 and S4) and we calculated the difference with the model median of the cost-optimal (default) phase-out year per region. This is not a perfect comparison, however, as Robiou du Pont et al.^25^ excluded LULUCF from the equity allocation calculations, whereas the cost-optimal scenarios included LULUCF. This difference could lead to earlier phase-out years in this study (on a global level: 10–20 years). The comparison showed that when taking a different equity approach, many of the countries studied here would have to phase out GHG emissions earlier than under a cost-optimal allocation, notably developed countries such as Canada and the EU, but also China. Brazil would be allowed to phase out emissions later, as well as other countries with lower per-capita emissions or developing economies, although with larger uncertainty (e.g., Indonesia). This implies that countries with later equity-based phase-out years could receive support from countries with earlier equity-based phase-out years, to help them meet their earlier domestic targets.

### Factors influencing the timing of the phase-out year

**Fig. 3:**
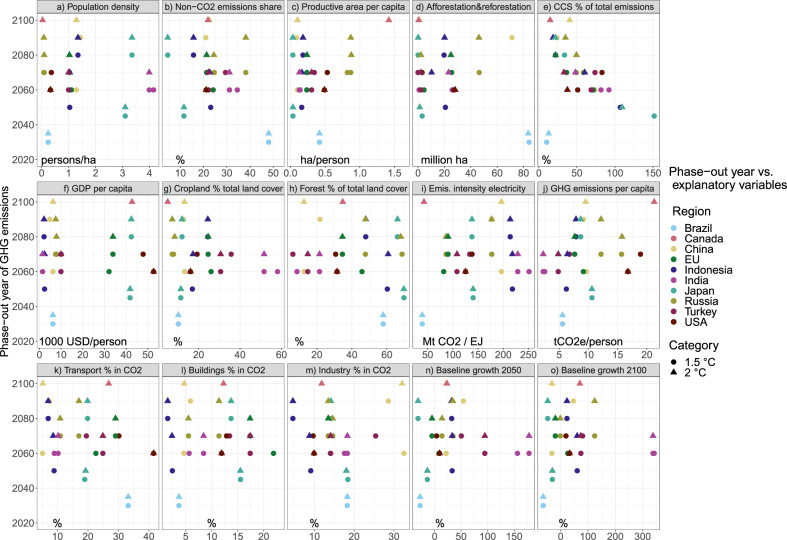
Fifteen explanatory variables vs. phase-out years across the ten countries (colours), the POLES and IMAGE models, and the 1.5 °C and 2 °C scenarios (shapes). See Supplementary Table 2 for details of how the variables were calculated (units are displayed in the lower left corner of each panel).

A key question is whether the different phase-out years can be explained. One would, for instance, expect the phase-out years for developed countries to be earlier than for developing countries, given the differences in baseline emission growth. However, Fig. 1 shows this is not consistently the case. We have, therefore, correlated the phase-out years with possible explanatory variables related to the mitigation potential. For this, we first selected 15 potentially explanatory variables as shown in Fig. 3 and listed in Supplementary Table 2 in Supplementary Methods and Results. To test for redundancy (internal correlation) in the dataset, the 15 factors were also used in a principal component analysis (PCA^26^, see Supplementary Methods and Results) to try and reduce the number of explanatory variables to the 5 most important ones. More detailed findings are provided in Supplementary Methods and Results (Supplementary Table 3 and Supplementary Fig. 4), as the PCA did not reveal clear patterns. Subsequently, Fig. 3 shows the relationship between each of the 15 variables and phase-out years across the 10 countries, 2 models (POLES and IMAGE) and the 2 scenarios (Supplementary Fig. 3 in Supplementary Methods and Results does so for all countries and models available in the dataset, for 1.5 °C and 2 °C separately). The IMAGE and POLES data subset was used to maximize the number of countries covered (and thereby the number of records as input to the statistical analyses), while ensuring the same number of models per country so as to not introduce a bias. Supplementary Fig. 6 shows that the six models in the full dataset show largely similar trends in emission-reduction pathways across regions, justifying the focus on two models here (Supplementary Fig. 7 shows that model differences are more pronounced for the share of solar and wind in electricity production, but not structurally explaining different phase-out years). Having different models per country makes it more difficult to distinguish clear patterns in the relationship between explanatory variables and phase-out years, but it is clear that some variables are indeed correlated with the phase-out year.

Finally, we used multiple linear regression. Different models to explain national phase-out years under 1.5 °C and 2 °C scenarios were tested, based on all possible combinations of four, five, six and seven variables (Supplementary Table 5). Supplementary Tables 6 and  7 in Supplementary Methods and Results show the results for these multiple linear regression models. Six turned out to be the optimal number of variables (see “Methods”). The model would then be (uncertainty range indicates two times SE):

1 $$y_i 	= 2079\left[ { \pm 6.7} \right] - 18.0\left[ { \pm 7.4} \right] \ast {\mathrm{CCSshare}} - 12.3\left[ { \pm 10.0} \right] \ast {\mathrm{Afforestation}} \\ 	\quad- 22.6\left[ { \pm 13.6} \right] \ast {\mathrm{transportshare}} + 13.7\left[ { \pm 11.9} \right] \ast {\mathrm{nonCO}}_2{\mathrm{share}} \\ 	\quad+ 20.9\left[ { \pm 16.8} \right] \ast {\mathrm{GDPcap}} - 6.5\left[ { \pm 7.1} \right] \ast {\mathrm{forestshare}} + \varepsilon _i$$

Where CCSshare stands for CO_2_ uptake from CCS as share of net total GHG emissions in 2050, Afforestation refers to CO_2_ uptake from afforestation and reforestation in 2050, transport share is the share of transportation emissions in total CO_2_ emissions in 2015, nonCO_2_share is the share of non-CO_2_ emissions in total GHG emissions in 2015, GDPcap is the gross domestic product (GDP) per capita in 2015, and forestshare is the share of forests in total land cover in 2015. A more parsimonious (simpler) model would contain only the variables with p-value smaller than 0.05, i.e. without forestshare. That model has slightly lower explanatory power, but the benefit is having further reduced the number of explanatory variables. The formula for the final model then becomes:

2 $$y_i 	= 2079\left[ { \pm 7.0} \right] - 18.7\left[ { \pm 7.6} \right] \ast {\mathrm{CCSshare}} - 16.3\left[ { \pm 9.4} \right] \ast {\mathrm{Afforestation}} \\ 	\quad- 20.1\left[ { \pm 13.9} \right] \ast {\mathrm{transportshare}} + 15.5\left[ { \pm 12.2} \right] \ast {\mathrm{nonCO}}_2{\mathrm{share}} \\ 	\quad+ 17.6\left[ { \pm 17.0} \right] \ast {\mathrm{GDPcap}} + \varepsilon _i$$

The signs can be explained as follows: the larger the CCS capacity and afforestation, the more potential for negative emissions contributing to faster reductions and an earlier phase-out year. The higher the current share of non-CO_2_ emissions, the more difficult to decarbonize so the later the phase-out. In addition, the higher the GDP per capita, the stronger the growth in emissions; thus, ceteris paribus, the later the phase-out. A higher GDP per capita could also imply greater capacity or willingness to mitigate emissions, but we only look at the default, cost-optimal case here, excluding equity considerations. The share of transport emissions showing a negative correlation is less straightforward. It seems to imply that this sector is relatively easy to decarbonize, which may hold for passenger transport, but not for freight and also not for international aviation. However, countries with a relatively large share of transport emissions often also have a relatively high GDP and smaller baseline emissions growth. A large transport share could also imply slower growth of this sector and smaller shares of other, more difficult to decarbonize sectors. All of these factors would contribute to earlier phase-out.

### Breakdown of emissions in the phase-out year

**Fig. 4:**
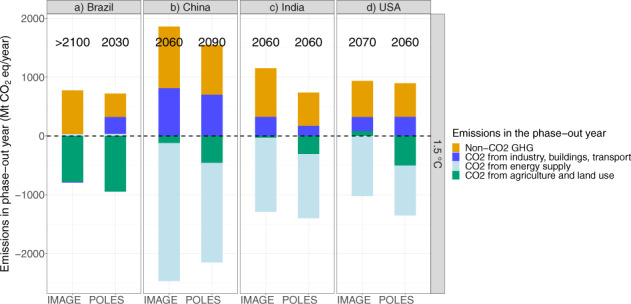
Breakdown of emissions in the phase-out year of total greenhouse gas emissions. Emissions in the phase-out year of GHG (year indicated per model—focusing on the same two models as in the previous section, for readability), by greenhouse gas (colours) and country (panels), focusing on a country with an average phase-out year (**b** China), a country with a late phase-out (**c** India), and two with an early projected phase-out of GHG emissions (**a** Brazil and **d** USA). Positive numbers denote remaining emissions of CH_4_, N_2_O and F-gases (non-CO_2_ GHG), and of CO_2_ in industry, buildings and transport, whereas negative numbers denote negative emissions in energy supply and in Agriculture, Forestry and Other Land Use (IPCC Category 3). CO_2_ from energy supply includes CO_2_ emissions from fuel combustion and fugitive emissions from fuels: electricity and heat production and distribution (IPCC category 1A1a), other energy conversion (e.g., refineries, synfuel production, solid fuel processing, IPCC category 1Ab, 1Ac), including pipeline transportation (IPCC category 1A3ei), fugitive emissions from fuels (IPCC category 1B) and emissions from carbon dioxide transport and storage (IPCC category 1C). Negative emissions in this sector result from the use of (BE)CCS.

It may also be possible to understand differences in phase-out years by looking at the different sources and sinks of emissions when net zero is achieved. Net zero means remaining emissions can be compensated by negative emissions elsewhere or in another sector. Figure 4 shows the emissions by GHG in the phase-out year. Results highlight that especially methane and N_2_O are hard to abate in most countries. In some models, also F-gases are a big source of remaining emissions in China and Japan, and to a smaller extent the United States. In developed and middle-income countries, the building sector forms a large share of the remaining CO_2_ emissions (this applies to the EU, China, Japan, the United States and, to some extent, to Russia). This is also true for the industry sector, although here some exceptions can be noted. The transport sector contributes to the remaining CO_2_ emissions in all countries studied here, except in Russia. In all countries except in Brazil, the energy supply sector is the largest contributor to negative CO_2_ emissions (through BECCS). Brazil, in contrast, is projected to realize most negative emissions through afforestation (see also ref. ^27^). Negative emissions through afforestation play a role in many other countries, but not so much in Japan, Canada and Russia. The POLES model projects more negative emissions from afforestation than IMAGE, contributing to its generally earlier phase-out, because it uses the inventory data. Some models project negative emissions in the industry sector in Brazil, Russia, Canada and, to a smaller extent, in the EU. Supplementary Table 9 shows the total negative emissions in 2100, which range from 188 Mt CO_2_ in Turkey to 2951 Mt CO_2_ in the USA, amounting to 22.4 Gt CO_2_ globally under the 1.5 °C scenario.

It should be noted that Brazil presents an exception for many indicators, as it has a relatively large share of non-CO_2_ emissions but an early phase-out. This can be explained by the breakdown of emissions in the phase-out year, which shows that a large potential for negative emissions can compensate for those remaining emissions. Other countries with an early phase-out (USA) generally also have a relatively large potential for negative emissions. Countries with a late phase-out (India and, to some extent, China and the EU) have relatively large remaining emissions of both CO_2_ and non-CO_2_ GHGs.

## Discussion

We analysed when major emitting countries are projected to reach CO_2_ and GHG emissions neutrality using 1.5 °C and 2 °C scenarios from IAMs. We also looked into the question how this depends on definitions and the reasons behind differences between countries.

In cost-optimal scenarios, Brazil, the United States (CO_2_ and all GHGs) and Japan (GHG only) are projected to have an earlier phase-out year than the global average. In contrast, India and Indonesia typically have a late phase-out year. For China, the EU and Russia, the phase-out year is typically near the global average. For several countries, the position vs. the global average is different for CO_2_ and all GHGs, and the specific climate target. The model spread is fairly large for Brazil and India, and to a smaller extent China, making these results less certain, and is smaller for the United States and the EU.

Definition factors (such as harmonization of data in the base year and the allocation of negative emissions) play a role in the phase-out year and this works out differently for different countries. These findings highlight the importance of clear definitions and political agreement on issues such as the use of land-use data and allocation of negative emissions. When harmonizing the model projections towards the countries’ reported net land-use emissions estimates in their GHG inventories, net-zero GHG emissions are projected to be reached earlier in all countries, except Brazil. The difference between inventory data and the model output for net land-use emissions is caused by a systematic difference in definition of anthropogenic land sources and sinks. As a result, inventory data are lower in all countries, except Brazil. The differences between these data sources are relatively large for China, India and the United States. When allocating negative emissions from biomass with CCS (BECCS) to the biomass-producing country instead of the carbon-storing country, phase-out years are earlier in Brazil, Indonesia, Canada, India and Russia (biomass producers, with a large model range for Brazil and India), but later in the EU, Japan and Turkey (importers). Updating GWPs from IPCC AR4 to IPCC AR5 values does not significantly affect phase-out years. Applying equity approaches rather than a cost-optimal allocation of mitigation effort would imply earlier phase-out years for many of the countries studied here, but later phase-out years for Brazil and other countries with lower per-capita emissions or developing economies (e.g., Indonesia, although with larger uncertainty).

The multiple linear regression showed that factors affecting negative emissions (e.g., afforestation and CCS) explain the lion’s share of the variance in phase-out years. Mitigation potential and especially the potential for negative emissions are dominant factors, determining when a country can reach net-zero emissions. Future CCS and afforestation capacity, as well as the current shares of transport emissions, non-CO_2_ emissions and GDP per capita, have the strongest relationship with phase-out years (negative for the former three, positive for the latter two). In addition to showing a relatively large potential for negative emissions, countries with a projected early phase-out (Brazil and the United States) generally have relatively low emission levels of CO_2_ from the energy demand sectors, a relatively high GDP per capita, low baseline growth, a low current share of non-CO_2_ emissions (except Brazil) and low population density.

That potential for negative emissions is high enough in Brazil to compensate for its relatively high levels of non-CO_2_ emissions, explaining the early phase-out. Countries with late phase-out (India and Indonesia, and to a smaller extent also China and the EU) show the reverse pattern and have relatively large remaining emissions of both CO_2_ and non-CO_2_ GHGs.

It should be noted that, so far, we focused on the outcomes of cost-optimal scenarios (using an equal marginal GHG price across all countries). In reality, national targets might also be based on equity principles^25,28^ (in line with the Paris Agreement’s common but differentiated responsibilities and respective capabilities). Therefore, Fig. 2d compares the results to those based on equity principles^25^. This has an impact on phase-out years. There are different ways to account for equity principles in international climate policy. Countries may choose to set different (in case of higher income countries more ambitious) domestic target years. Alternatively, it is also possible to use flexible instruments (emission trading, investments in other countries). The IAM results indicate mitigation measures that countries should implement domestically under a globally cost-optimal distribution. These results do not answer the question of how these measures are funded and how much effort or finance each country is providing. Equity frameworks can distribute the emissions of IAMs^25, 29, 30^. As such, this could still lead to the outcomes as described in this study. It does mean, however, that policy-makers should not simply use the phase-out years presented here to set national targets. This study can be seen as a first step to inform such target setting, but national models or other tools will need to be applied, to fully incorporate relevant domestic circumstances. That will need to include the country’s perspective of a national contribution to the global mitigation effort, also reflecting equity considerations, as well as account for the outcome of negotiations on Article 6 and international transfer of mitigation outcomes (ITMOs). As such, a country could implement an equitable emission target based on a combination of domestic targets (informed by IAMs and national models) and ITMOs. The Convention of the UNFCCC (1992) already states that climate policies should be cost-effective and equity considerations can be dealt with through, e.g., trading and financial support^31^. Further, the Paris Agreement recognizes that countries could make use of ITMOs. The national target setting can further be informed by studies on co-benefits such as ref. ^32^, which suggest a significant share of mitigation costs could be covered by accounting for air quality and other co-benefits, making additional domestic mitigation more attractive.

Another critical point is that the scenarios were created in the period 2016–2018. This implies that cost-optimal policies were assumed to be implemented from 2020 onwards. This means that in some countries (e.g., Brazil) the political reality is not likely to lead to the pathways as described in the models. On the other hand, many other countries have now adopted or announced net-zero emission targets. China’s announced 2060 carbon neutrality goal, the EU’s 2050 net-zero GHG goal, Japan’s announced 2050 net-zero GHG goal and the USA’s tentative 2050 net-zero GHG emissions goal (suggested in the Biden–Harris climate plan^33^) are all in line with the models’ domestic cost-optimal mitigation pathways for 2 °C and 1.5 °C, and in some cases are even more ambitious (e.g., rely less on negative emissions). Although several countries have announced net-zero emission goals, it should be noted that the (aggregated) impact of the NDCs seems insufficient to be on a pathway to meet these^34^. Canada’s foreseen 2050 net-zero emissions goal does not specify whether it would apply to all GHG or CO_2_ only, but both would need to be phased out slightly earlier than 2050 to be in line with the models’ cost-optimal 1.5 °C scenarios (for 2 °C, 2050 net-zero emissions would suffice according to these models). Either way, the specification of target coverage is important. Our findings show that to meet these targets, countries should pay special attention to enhancing the capacity to realize negative emissions, clearly specify the land-use emissions accounting and related data (especially important for Canada and the USA), agree on the accounting of negative emissions from BECCS (important for Brazil and Japan) and clarify their approach to equity and the use of ITMOs (all countries).

Future work could analyse a few other factors that affect national differences in phase-out years but that we did not consider here: metrics other than GWPs^6, 7^ and consumption-based vs. production-based emissions accounting^35^. It could further analyse more scenarios from more, different types of models (national, sectoral and macro-economic) for more countries. With such an enlarged dataset, a PCA would be more useful. Alternatively, one could dive into the results of one model and tease out underlying dynamics. A comparison of scenario results with countries’ submitted long-term strategies would further be useful: on the one hand, to identify additional mitigation potential for these strategies and, on the other hand, to make the scenarios better reflect political realities. That is also where social sciences could add value to this work: guide the social acceptance and practical implementation of net-zero targets, with an understanding of relevant actors and their motivations. Ongoing work on political feasibility of mitigation scenarios^36^, e.g., could shed light on governments’ capacity to implement net-zero targets.

Our results can inform the national target setting, as they present an advancement in knowledge on national-level results from IAM scenarios, as often used in IPCC assessments. The results notably address the Talanoa Dialogue questions of Where do we want to go? and How do we get there?. They can also inform international negotiations related to Article 6 and methodological choices, such as LUC data and accounting for negative emissions from BECCS. Furthermore, non-state actors can help their governments define realistic and potentially more ambitious targets.

## Methods

### Overall method

We used a set of scenarios from six IAMs to analyse the projected phase-out years for different countries. Subsequently, we applied a number of methods to determine which factors explain differences in phase-out years between countries. First, we made a selection of 15 variables that potentially explain why some countries see earlier phase-out and others later. Second, we tested for redundancy using PCA (see Supplementary Methods and Results) and visually inspected the data. Third, multiple linear regression was applied to select those variables with the strongest relation to phase-out year. This was required because of the limited number of records in the dataset: ten countries, two scenarios and six models with varying country coverage. This selection of variables best explaining phase-out year differences was constructed by trying out all 3003 possible combinations of 4, 5, 6 and 7 of the 15 variables in multiple linear regression, selecting those combinations resulting in the highest *R*^2^ (degree to which the data are explained by the model). We ended up with six variables, because it improved the *R*^2^ (as well as adjusted the *R*^2^ that penalizes having more explanatory variables) with respect to four and five variables, whereas selecting seven did not result in significant improvements (see Supplementary Table 5). In the multiple linear regression, we used standardized variables given their different units. We only used the projections by the POLES and IMAGE models for the multiple linear regression, because these are the only two models that cover all ten countries. Therefore, that data subset had an equal number of records for each country (i.e., four : two scenarios for each model), while still representing more than one model for robustness.

### Scenario data

The analysis presented here uses the scenario projections of the six models from a multi-model study^9, 37^ using the same protocol for reaching a cost-optimal pathway to adhere to global carbon budgets of 1000 and 400 Gt CO_2_ for the 2011–2100 period, allowing temporal overshoot. The two budgets represent limiting global warming to below 2 °C during the twenty-first century and below 1.5 °C in 2100 with more than 66% probability. In the scenarios, cost-optimal mitigation was assumed to start in 2020 (i.e., emission reductions where and when they are cheapest to achieve). Up to 2020, it was assumed that only existing policies were implemented (historical data up to 2020 was not yet available when these scenarios were developed between 2016 and 2018). Non-CO_2_ emissions were taxed with the same carbon price as that of CO_2_ in the cost-optimal scenarios.

The regional coverage of the models differs (see Supplementary Table 8 in Supplementary Methods). For some countries, therefore, the results are based on a lower number of models (with obvious consequences for certainty of the results, we indicated the number of models per country). In some cases, the existing model output was made more comparable with the country definitions used in this study (see Supplementary Table 8). Results are shown for ten selected major emitting economies, i.e., Brazil (covered by three out of six models), Canada (three), China (six), EU (six; it is noteworthy that all projections for the EU in this study include the United Kingdom), India (six), Indonesia (three), Japan (four), Russia (three), Turkey (three), and USA (six), representing two-thirds of the global GHG emissions including land-use change and international transport emissions in 2018^38, 39^.

Emission pathways for the ten countries were linearly extrapolated to 2200 based on the 2050–2100 trajectory, to estimate the phase-out years beyond 2100 where needed. We used the CO_2_-equivalent emissions based on GWPs from IPCC AR4 (time horizon of 100 years) as default and show the effect of using those from AR5. The text of the Paris Agreement leaves the choice of metric open and refers to the common metrics assessed by the IPCC.

For the equity-sensitivity analysis in Fig. 2d, we used phase-out years directly from Robiou du Pont et al.^25^. They based their equity calculations on 2 °C and 1.5 °C scenarios from the IPCC AR5 database and on PRIMAP data for historical and projected population, GDP and GHG emissions to model country allocations under different equity approaches. The parameterization of the equity approaches follows Robiou du Pont et al.^40^.

## Supplementary information

Supplementary Information

## Data Availability

Model results can be found in the open access CD-LINKS scenario explorer https://data.ene.iiasa.ac.at/cd-links/. Policy-relevant data are available in the Global Stocktake tool https://themasites.pbl.nl/o/global-stocktake-indicators/.

## Code availability

The code from the six integrated assessment models is not available in a publicly shareable version, although several have published open source code, visualization tools or detailed documentation (see references). The R-script that was used to generate the figures^41^ can be found on GitHub https://github.com/CD-LINKS/factsheet/tree/master/src/Neutrality and on Zenodo https://zenodo.org/badge/latestdoi/57221386.
