# Supplementary material for: Net-zero emission targets for major emitting countries consistent with the Paris Agreement
Source: Nat Commun. 2021 Apr 9;12:2140. doi: 10.1038/s41467-021-22294-x (PMC8035189; doi:10.1038/s41467-021-22294-x)
Supplement: Supplementary file 1 — Supplementary Information [file 41467_2021_22294_MOESM1_ESM.pdf]

Supplementary Information

Net-zero emission targets for major emitting countries consistent with the Paris Agreement

Heleen L. van Soest[1,2]*, Michel G. J. den Elzen[1], Detlef P. van Vuuren[1,2]

[1] PBL Netherlands Environmental Assessment Agency, P.O. Box 30314, 2500 GH The Hague, The Netherlands

[2] Copernicus Institute of Sustainable Development, Utrecht University, P.O. Box 80.115, 3508 TC Utrecht, The Netherlands

*Corresponding author:

Heleen L. van Soest

Heleen.vanSoest@pbl.nl

Supplementary Methods and Results: Emission pathways and the influence of definitions

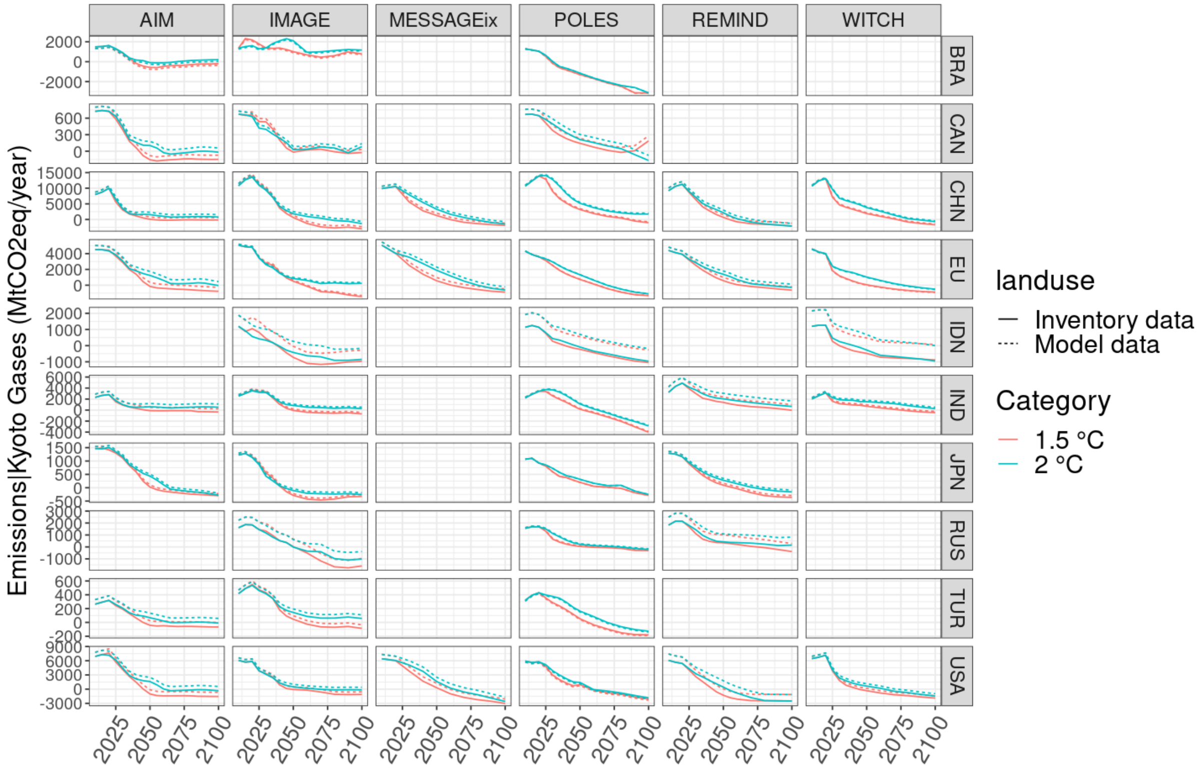

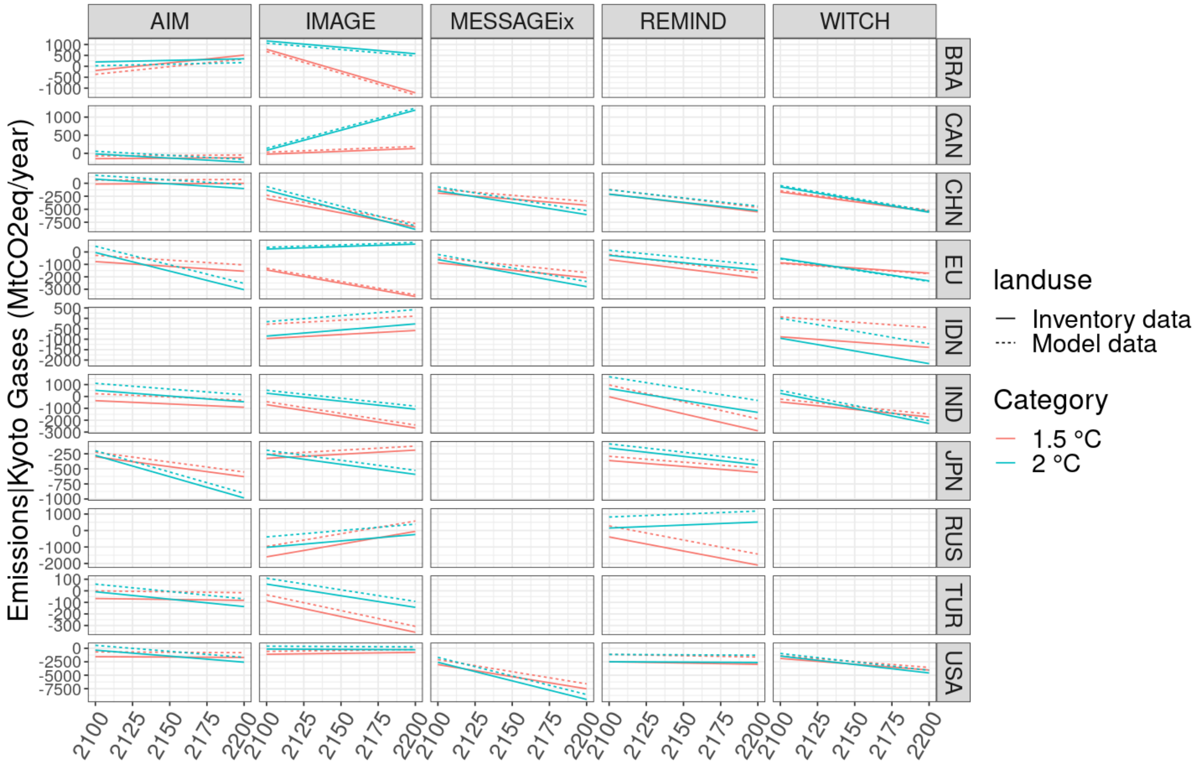

Supplementary Figure 1: GHG emissions pathways when using model land-use change data versus when using inventory LULUCF data. GHG emissions pathways ($MtCO_2eq$ per year) when using model land-use change (LUC) data (dotted lines) versus when using inventory LULUCF data (solid lines), in 1.5 °C (pink) and 2 °C (blue) scenarios, per model (horizontal panels) and country (vertical panels), for the time period 2010-2100 (upper graph) and the time period 2100-2200 (lower graph, extrapolation). For the solid lines, we calculated the difference between a model's 2010 LUC CO_2 emissions and the inventory's 2010 LULUCF CO_2 emissions (per country); subtracting that offset from the model LUC CO_2 projections (offset harmonisation method); adding the adjusted LULUCF CO_2 emissions projections to the model's projections for GHG emissions excluding LUC CO_2; and finally, calculating the phase-out year for the adjusted emission pathway. POLES was excluded from the lower graph as it does not show a significant difference between model data and inventory data.

Supplementary Table 1: phase-out year of Kyoto GHG emissions with model LUC data versus inventory LULUCF data. NA: no phase-out

Model	Scenario	Country	Phase-out year with model LUC data	Phase-out year with inventory LULUCF data
**IMAGE 3.0**	1.5 °C	Canada	NA	2050
**AIM V2.1**	1.5 °C	China	NA	2055
**IMAGE 3.0**	1.5 °C	Mexico	NA	2060
**IMAGE 3.0**	2 °C	USA	NA	2070
**POLES CDL**	1.5 °C	Canada	NA	2080
**IMAGE 3.0**	2 °C	Canada	NA	2090
**AIM V2.1**	2 °C	India	NA	2154
**IMAGE 3.0**	2 °C	Brazil	NA	NA
**POLES CDL**	2 °C	China	NA	NA
**IMAGE 3.0**	2 °C	EU	NA	NA
**IMAGE 3.0**	2 °C	Mexico	NA	NA
**REMIND-MAgPIE 1.7-3.0**	2 °C	Russia	NA	NA

Historical LULUCF emissions data sources

For the selected Annex I countries (Canada, the European Union, Japan, the Russian Federation, Turkey and the USA), we used the GHG inventories submitted in 2019 to the UNFCCC[1]. For historical emissions for non-Annex I Parties, the data was taken from the UNFCCC GHG databases[2], in which the GHG inventory data reported in most recent Biennial Update Reports (BURs) submitted to the UNFCCC were compiled. The sources are described in detail in Kuramochi et al.[3]. National Inventory Reports (NIR) and National Communications (NC) were also used for some countries. For Brazil, the emissions inventory from Sistema de Estimativa de Emissões de Gases de Efeito Estufa[4] was used.

Allocation of negative emissions from BECCS

Emissions|CO2|Allocation was used to calculate the phase-out year of CO_2 emissions when negative emissions are allocated, ex-post, to the biomass producer instead of the carbon-storing country. *Emissions|CO2|Allocation,* for country i (where w stands for world) and timestep t, was calculated as:

$$Emissions|CO2|Allocation_{i,t} = Emissions|CO2_{i,t} + CCS|Biomass_{i,t} - (Agricultural\ Production|Energy_{i,t} / Agricultrual\ Production|Energy_{w,t}) * CCS|Biomass_{w,t}$$

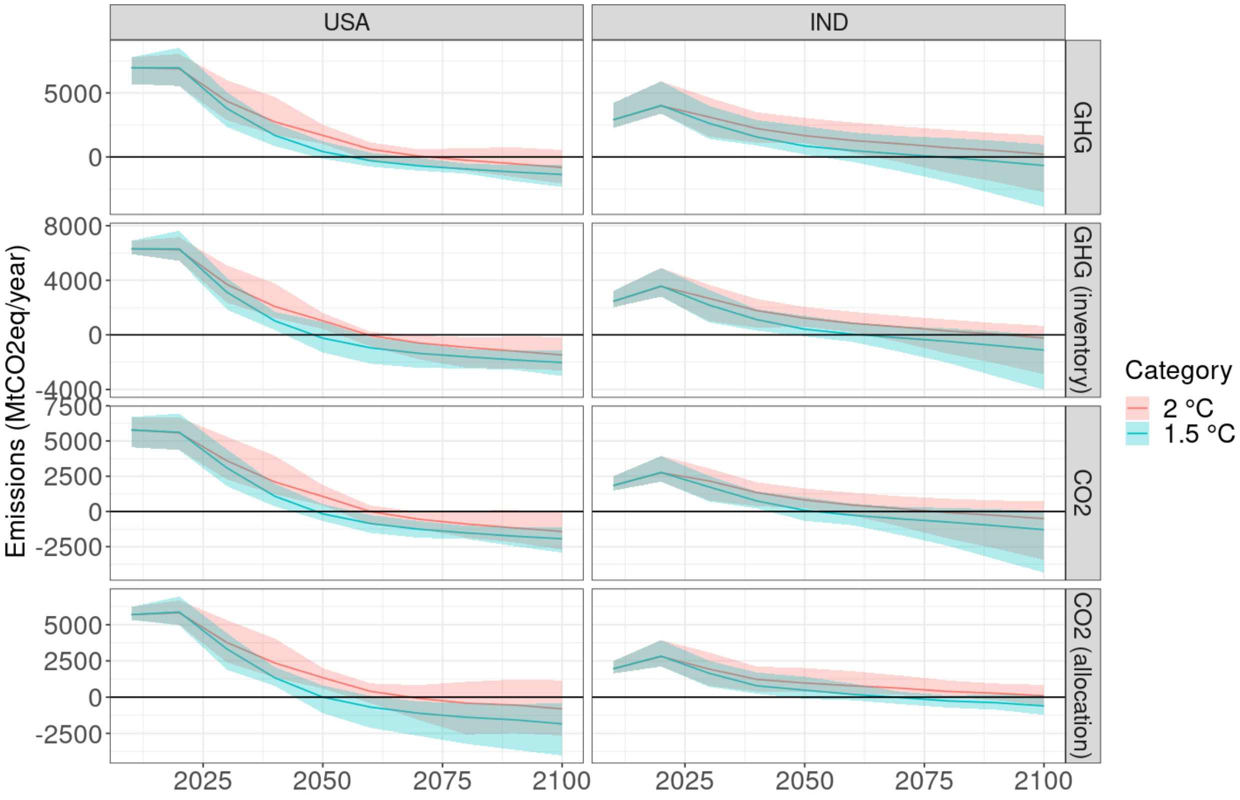

Supplementary Figure 2: Emission pathways for the USA and India under the default and sensitivity cases. Emission pathways ($MtCO_2eq$) for a country with an early phase-out (USA) and a country with a late phase-out (India), for 2 °C and 1.5 °C scenarios, for the default case of total Kyoto GHG emissions ('GHG'), GHG emissions when using inventory LULUCF data ('GHG (inventory)'), CO_2 emissions ('CO_2'), and CO_2 emissions when allocating negative emissions from BECCS to the biomass producer ('CO_2 (allocation)'). Lines indicate model mean, funnels indicate the range (minimum – maximum).

Supplementary Methods and Results: Multiple linear regression and Principal Component Analysis

Additional information on variables and scatter plots

Supplementary Table 2: Variables used in multiple linear regression and principal component analysis, abbreviations used in text and figures, details of calculation and unit. Variables are grouped: grey – underlying driver, orange – current build-up of energy system and emissions (indication of hard-to-abate sectors) , light green – current indications of potential for negative emissions, dark green – future potential for negative emissions.

Variable	Abbreviation	Details	Unit
GDP per capita 2015	Gdpcap	2015 value for GDP|MER / Population	1000 USD/person
GHG emissions per capita 2015	Emiscap	2015 value for Emissions|Kyoto Gases / Population	tCO_2e/person
Growth of GHG emissions in baseline 2050	BaselineGHG2050	2050 value for Emissions|Kyoto Gases (2050 – 2015) / 2015 *100	%
Growth of GHG emissions in baseline 2100	BaselineGHG2100	2100 value for Emissions|Kyoto Gases (2100 – 2015) / 2015 *100	%
Transport share in total CO_2 emissions 2015	Transportshare	2015 value for Emissions|CO2|Energy|Demand|Transportation / Emissions|CO2	%
Buildings share in total CO_2 emissions 2015	Buildingshare	2015 value for Emissions|CO2|Energy|Demand|Residential and Commercial / Emissions|CO2	%
Industry share in total CO_2 emissions 2015	Industryshare	2015 value for Emissions|CO2|Energy|Demand|Industry / Emissions|CO2	%
Emissions intensity electricity sector 2015	Emisint	2015 value for Emissions|CO2|Energy|Supply|Electricity / Secondary Energy|Electricity	Mt CO_2 / EJ
Non-CO_2 share 2015	nonCO2share	2015 value for (N_2O in CO_2eq + CH_4 in CO_2eq + F-gases in CO_2eq) / Kyoto Gases	%
Population density 2015	Density	2015 value for Population / Land cover	Persons/ha
Productive area per capita 2015	Prodcap	2015 value for Land Cover|Cropland / Population	ha/person
Cropland share of total land cover 2015	Cropshare	2015 value for Land Cover|Cropland / Land Cover	%
Forest share of total land cover 2015	Forestshare	2015 value for Land Cover|Forest / Land Cover	%
Carbon Sequestration|CCS 2050	CCSshare	2050 value for Carbon Sequestration|CCS / abs(Kyoto Gas emissions + Carbon Sequestration|CCS)	%
Land Cover|Forest| Afforestation and Reforestation 2050	Afforestation	2050 value for Land Cover|Forest| Afforestation and Reforestation	Million ha

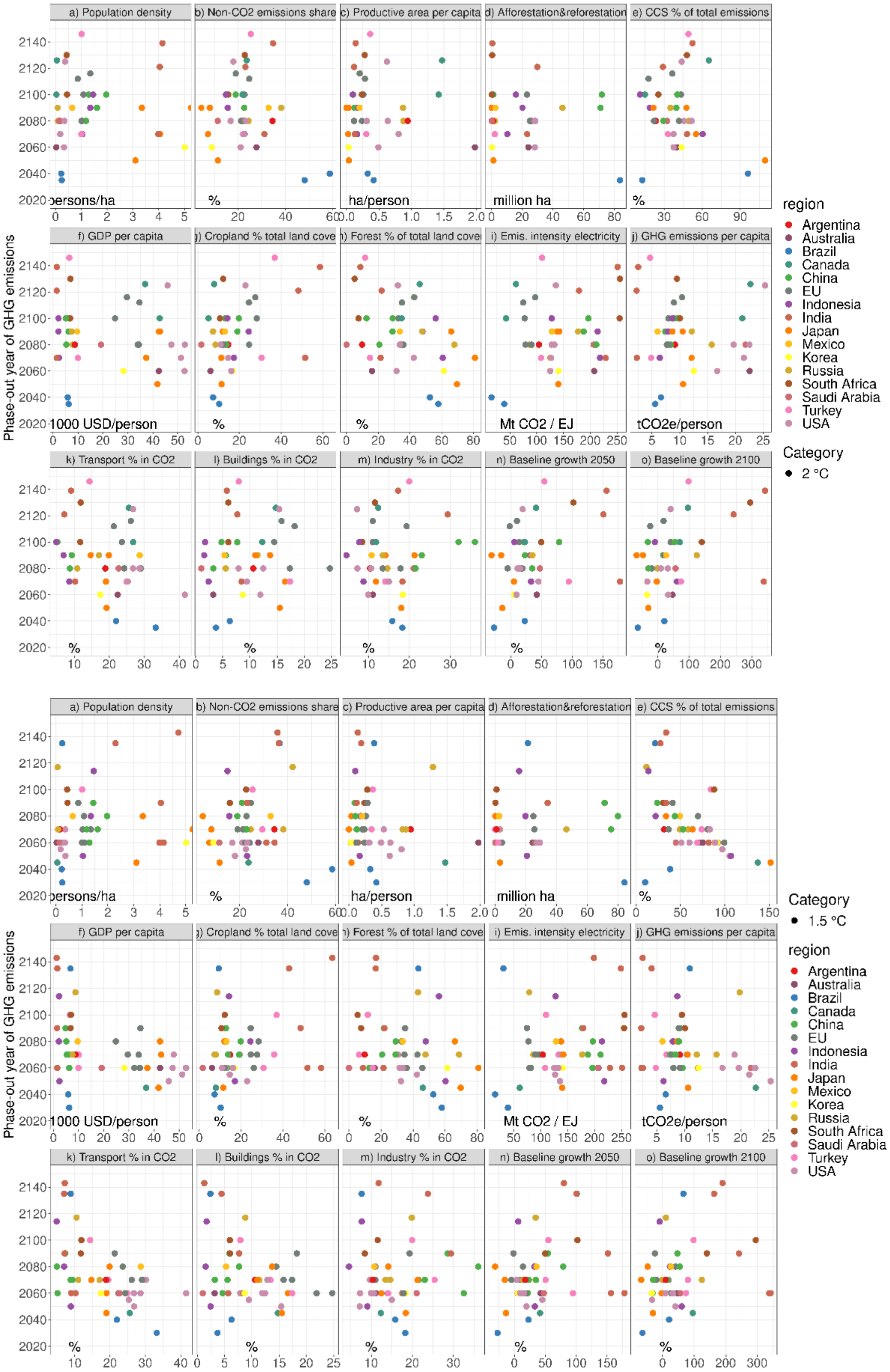

Supplementary Figure 3: Phase-out years as function of explanatory variables. 15 explanatory variables versus phase-out years across all countries available in the dataset (colours, noting different model coverage per country), all models and the 2 °C scenario

(upper graph) and 1.5 °C scenario (lower graph). See Supplementary Table 2 for details of how the variables were calculated and their units.

Methods: PCA

The dataset, consisting of projections by six models for 15 variables, for a number of countries (different per model), for both 1.5 °C and 2 °C scenarios, was further refined for the PCA:

- Only models with complete reporting (all 15 variables) were used. Missing land cover data for Japan by the REMIND model and missing afforestation data for AIM and REMIND meant REMIND and AIM had to be removed for the PCA to work.
- MESSAGE data for 2010 and 2020 were interpolated to get an estimate for 2015.
- Different subsets of the dataset described above were created for sensitivity analysis on the limited number of records (countries), consisting of:
    - All models (despite varying country coverage), both 1.5 °C and 2 °C scenarios, and ten countries;
    - All models, both 1.5 °C and 2 °C scenarios, 10 countries, but excluding those with no projected phase-out;
    - Model median (noting that for each country, a different number of models was available), both 1.5 °C and 2 °C scenarios, and 10 countries;
        - Model median, only 1.5 °C scenarios, and 10 countries;
        - Model median, only 2 °C scenarios, and 10 countries;
    - Only the POLES and IMAGE models (as both cover all 10 countries), both 1.5 °C and 2 °C scenarios, and 10 countries;
    - Only the POLES model, both 1.5 °C and 2 °C scenarios, and 16 countries (to increase the number of records; only POLES covers these 16 countries).
- For POLES, forest cover data for Saudi Arabia was set at 0 due to a reporting error.

Results: PCA

The scree plot (Supplementary Figure 5) does not show a strong decline, so we only used the PCA for the corroboration of the results.

The first principal component explains 37% of the variance in national phase-out years. Its largest contributors are baseline growth, emissions per capita, emissions intensity of electricity, and the share of cropland area for biomass (Supplementary Table 3). The second principal component explains 17% of the variance, and has the largest contributions from non-CO_2 emissions share, afforestation, CCS share, building sector emissions share, and GDP per capita. The third principal component explains another 13% of the variance, and the fourth 8%, for a cumulative proportion of explained variance of 75% for the first four principal components.

Applying the proportion of variance as weighting factor (i.e. absolute contribution of variable to PC1 times proportion of variance explained by PC1, plus absolute contribution of variable to PC2 times proportion of variance explained by PC2, etc.), resulted in the following top five explanatory variables: **productive area per capita, GDP per capita, buildings emissions share, transport emissions share, and emissions intensity of electricity.** This list differs from the multiple linear regression results due to the different purposes, with

PCA mainly aiming to explain the variance in the input data, by reducing redundancy in the dataset (correlated variables). The PCA was also performed on the other data subsets, for sensitivity analysis. The top five explanatory variables for each of these is shown in Supplementary Table 4.

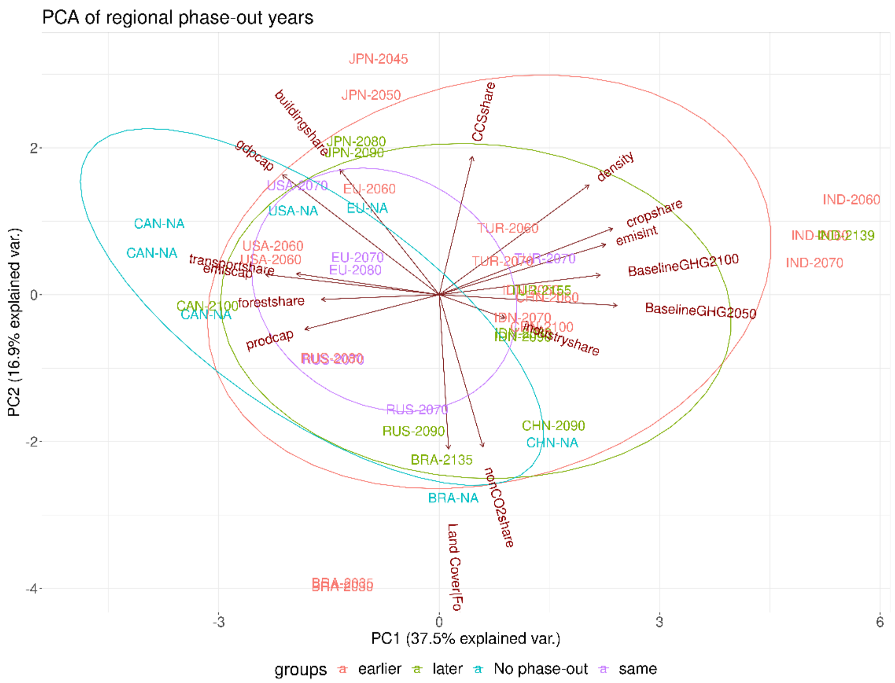

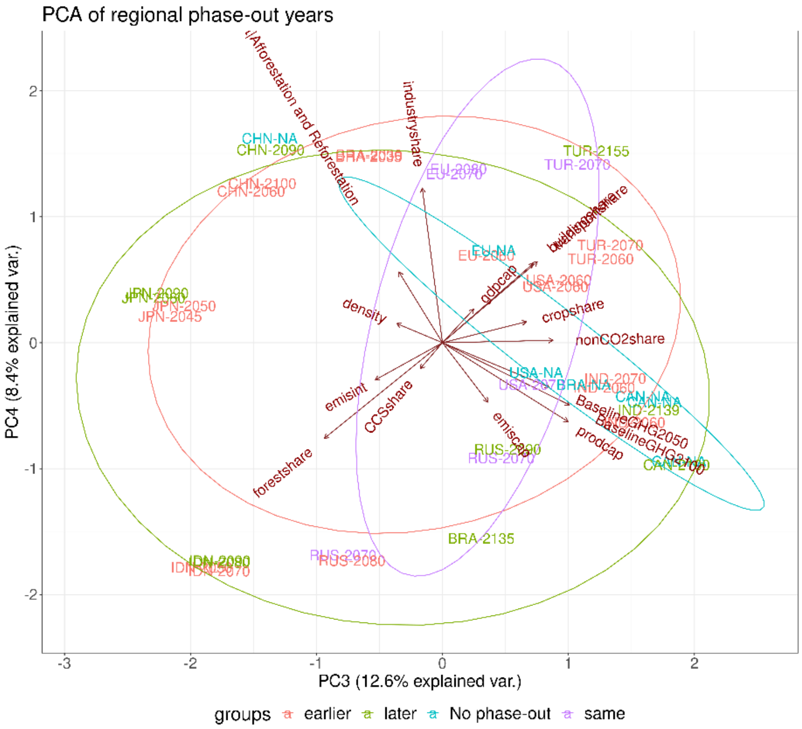

Supplementary Figure 4: Principal Component Analysis of the national phase-out years of GHG emissions. Principal Component Analysis of the national phase-out years of GHG emissions, for both 1.5 °C and 2 °C scenarios by the POLES and IMAGE models. The upper graph shows PC1 and PC2, while the lower graph shows PC3 and PC4. Arrows indicate the contribution of each component to each PC (i.e., loading). Coloured circles group countries with a phase-out year before the global average (pink), similar to the

global average (purple), later than the global average (green), and without a phase-out (blue). Individual countries are indicated by their three-letter codes plus phase-out year (multiple entries result from having two models and two scenarios).

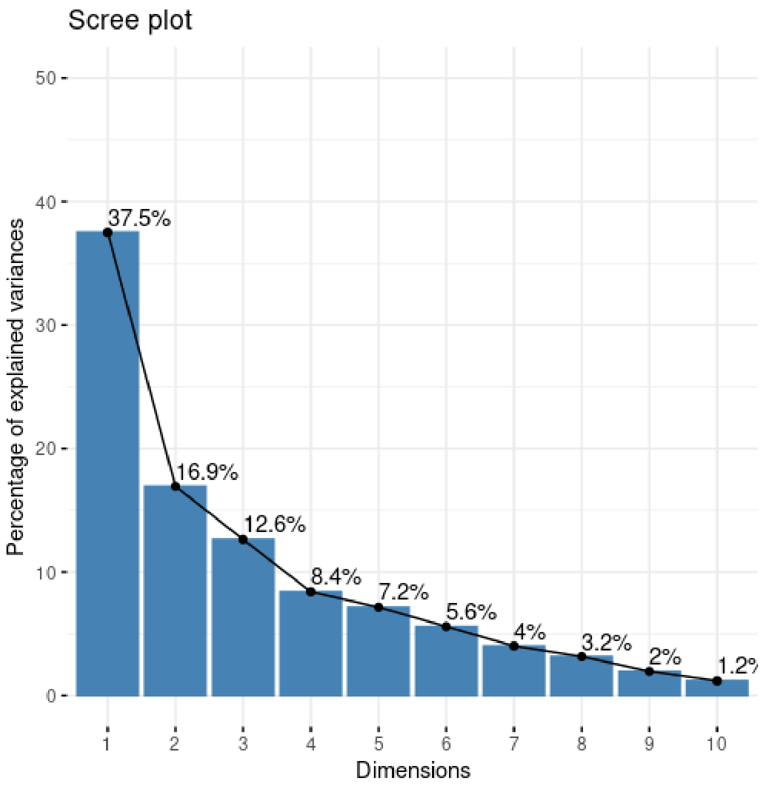

Supplementary Figure 5: Scree plot for the PCA using the 'IMAGE and POLES' dataset.

Supplementary Table 3: Contribution of each explanatory variable to the principal components (loadings), and summary statistics of the principal component analysis using the 'IMAGE and POLES' dataset.

Rotation	PC1	PC2	PC3	PC4	PC5	PC6	PC7	PC8	PC9	PC10	PC11	PC12	PC13	PC14	PC15
**Population density 2015**	0.29	0.32	-0.14	0.07	-0.16	0.07	-0.02	0.54	-0.34	0.10	-0.09	0.06	-0.37	0.36	0.24
**Non-CO2 share 2015**	0.09	-0.44	0.34	0.01	-0.06	0.45	0.07	-0.13	-0.29	0.22	0.46	-0.19	-0.16	0.21	-0.04
**Productive area per capita 2015**	-0.26	-0.10	0.39	-0.30	0.29	0.07	-0.11	0.29	0.18	0.36	-0.35	0.25	-0.27	0.05	-0.28
**Carbon Sequestration|CCS 2050**	0.02	-0.45	-0.13	0.27	-0.27	0.15	-0.58	0.23	0.29	-0.25	-0.19	-0.18	-0.09	0.03	-0.07
**Land Cover|Forest|Afforestation and Reforestation 2050**	0.07	0.40	-0.07	-0.10	0.20	0.67	-0.26	-0.40	-0.01	-0.20	-0.22	0.01	-0.14	-0.03	0.01
**GDP per capita 2015**	-0.31	0.35	0.10	0.13	-0.14	-0.11	-0.24	0.13	-0.36	-0.11	0.25	-0.18	-0.13	-0.27	-0.57
**Cropland share of total land cover 2015**	0.34	0.19	0.26	0.08	-0.23	-0.03	0.07	-0.10	0.07	0.35	-0.41	-0.53	0.27	0.08	-0.21
**Forest share of total land cover 2015**	-0.23	-0.01	-0.36	-0.36	-0.21	0.43	0.13	0.36	-0.02	0.12	0.05	-0.01	0.52	-0.01	-0.13
**Emissions intensity electricity sector 2015**	0.33	0.15	-0.21	-0.14	0.05	-0.16	-0.50	-0.14	0.22	0.39	0.41	0.23	0.13	0.20	-0.18
**GHG emissions per capita 2015**	-0.34	0.06	0.14	-0.22	0.31	-0.19	-0.36	0.04	-0.19	-0.11	0.03	-0.41	0.25	0.38	0.35
**Transport share in total CO2 emissions 2015**	-0.28	0.06	0.29	0.30	-0.42	0.05	-0.20	-0.19	-0.22	0.16	-0.16	0.49	0.29	0.10	0.20
**Buildings share in total CO2 emissions 2015**	-0.19	0.36	0.28	0.30	-0.03	0.18	0.17	0.20	0.62	-0.08	0.35	-0.07	0.02	0.18	0.10
**Industry share in total CO2 emissions 2015**	0.13	-0.07	-0.06	0.58	0.61	0.14	-0.02	0.23	-0.18	0.12	-0.03	0.07	0.34	-0.11	-0.09
**Growth of GHG emissions in baseline 2050**	0.35	-0.03	0.33	-0.17	0.02	-0.04	0.06	0.10	-0.06	-0.59	-0.01	0.26	0.27	0.33	-0.34
**Growth of GHG emissions in baseline 2100**	0.31	0.06	0.39	-0.23	-0.04	0.06	-0.23	0.27	0.00	-0.03	0.14	0.01	0.12	-0.63	0.36

Summary	PC1	PC2	PC3	PC4	PC5	PC6	PC7	PC8	PC9	PC10	PC11	PC12	PC13	PC14	PC15
**Standard deviation**	2.37	1.59	1.38	1.12	1.04	0.92	0.78	0.69	0.54	0.42	0.32	0.26	0.17	0.10	0.08
**Proportion of variance**	0.37	0.17	0.13	0.08	0.07	0.06	0.04	0.03	0.02	0.01	0.01	0.00	0.00	0.00	0.00
**Cumulative proportion**	0.37	0.54	0.67	0.75	0.83	0.88	0.92	0.95	0.97	0.99	0.99	1.00	1.00	1.00	1.00

Supplementary Table 4: Top five explanatory variables for each of the seven data subsets used in PCA.

Rank (weighted)	All models, 1.5 and 2 °C, 10 countries	All models, 1.5 and 2 °C, 10 countries, exclude 'no phase-out' countries	Only POLES and IMAGE, 1.5 and 2 °C, 10 countries	Only POLES, 1.5 and 2 °C, 20 countries	Model median, 1.5 and 2 °C, 10 countries	Model median, 1.5 °C, 10 countries	Model median, 2 °C, 10 countries
**1**	Transportshare	Transportshare	Prodcap	Density	BaselineGHG2100	Transportshare	emisint
**2**	BaselineGHG2100	Prodcap	GDPcap	emisint	Density	Density	BaselineGHG2100
**3**	Forestshare	Forestshare	Buildingshare	Emiscap	Transportshare	BaselineGHG2100	Buildingshare
**4**	GDPcap	Density	Transportshare	Prodcap	Cropshare	Cropshare	Prodcap
**5**	Prodcap	Cropshare	emisint	CCSshare	Prodcap	Forestshare	Cropshare

Results: regression

Supplementary Table 5: Results for the multiple linear regression, after trying all possible combinations of four, five, six or seven variables on the dataset containing both 1.5 °C and 2 °C scenarios, and selecting the one with highest R-squared (displayed here). Adjusted R-squared is also provided, as well as the variables.

	R-squared	Adjusted R-squared	Variables
**Combination of 4**	0.53	0.46	Afforestation<br>CCSshare<br>forestshare<br>transportshare
**Combination of 5**	0.58	0.50	nonCO2share<br>afforestation<br>CCSshare<br>gdpcap<br>transportshare
**Combination of 6**	0.63	0.54	nonCO2share<br>afforestation<br>CCSshare<br>gdpcap<br>forestshare<br>transportshare
**Combination of 7**	0.63	0.53	nonCO2share<br>afforestation<br>CCSshare<br>gdpcap<br>forestshare<br>transportshare<br>buildingshare

Supplementary Table 6: Detailed results for the multiple linear regression, for the combinations of five and six variables. Weight and significance (p-value) per variable are provided.

Model: phase-out year versus	R-squared	p-value	Variables	Weight [significance]
Combination of 5 variables	0.58	0.0002	Afforestation<br>NonCO2share<br>CCSshare<br>Gdpcap<br>Transportshare	-16.3 [0.002]<br>15.5 [0.02]<br>-18.7 [0.00004]<br>17.6 [0.05]<br>-20.1 [0.007]
Combination of 6 variables	0.63	0.0001	Afforestation<br>NonCO2share<br>CCSshare<br>GDPcap<br>Transportshare<br>Forestshare	-12.3 [0.02]<br>13.7 [0.03]<br>-18.0 [0.00004]<br>20.9 [0.02]<br>-22.6 [0.003]<br>-6.5 [0.08]

Supplementary Table 7: multiple linear regression results for 1.5 °C and 2 °C separately. Although higher R-squared may be obtained when using only 1.5 °C or only 2 °C scenarios, selecting only one scenario halves the number of records in the dataset, which was already relatively small, thereby decreasing the reliability of the results. In addition, one would want to be able to explain different phase-out years across countries and across all 'Paris-consistent' scenarios, not just for one specific scenario.

	1.5 °C				2 °C			
Model: phase-out year versus	R-squared	p-value	Variables	p-value	R-squared	p-value	Variables	p-value
Combination of 5 variables	**0.84**	0.0002	Density<br>Transport%<br>CCSshare<br>Gdpcap<br>Baseline2050	0.004<br>0.00008<br>0.0004<br>0.0001<br>0.0008	**0.90**	0.0003	Baseline2100<br>Forestshare<br>Afforestation<br>GDPcap<br>Baseline2050	0.0004<br>0.0003<br>0.0002<br>0.001<br>0.0003
Combination of 6 variables	**0.88**	0.0002	Density<br>CCSshare<br>Gdpcap<br>emisint<br>Transportshare<br>BaselineGHG2050	0.01<br>0.0009<br>0.0001<br>0.08<br>0.00003<br>0.0004	**0.93**	0.0004	Afforestation<br>CCSshare<br>Gdpcap<br>Forestshare<br>BaselineGHG2050<br>BaselineGHG2100	0.0001<br>0.11<br>0.001<br>0.0009<br>0.0003<br>0.0003

Supplementary Methods: Overview of models per country

Supplementary Table 8: Overview of models and covered countries for total GHG emissions, after scenario selection, i.e. only including models with projections up to 2100. X indicates availability of both 1.5 °C and 2 °C scenarios.

Country	Number of models	AIM V2.1	IMAGE 3.0	MESSAGEix_GLOBIOM_1.1	POLES CDL	REMIND-MAgPIE 1.7-3.0	WITCH2016
**Brazil**	3	X	X	-	X	-	-
**Canada**	3	X	X	-	X	-	-
**China**	6	X	X	X	X	X	X
**EU**	6	X	X	X [1]	X	X	X
**India**	6	X	X	-	X	X	X
**Indonesia**	3	-	X	-	X	-	X
**Japan**	4	X	X	-	X	X	-
**Russia**	3	-	X	-	X	X	-
**Turkey**	3	X	X	-	X	-	-
**USA**	6	X	X	X [2]	X	X	X
**World**	6	X	X	X	X	X	X

[1] *The EU-projections by the MESSAGE model were adjusted to exclude Turkey (by subtracting emissions projections for Turkey by the IMAGE model)*

[2] *USA projections by the MESSAGE model were adjusted to exclude Canada (based on IMAGE projections).*

Supplementary Results: Additional indicators

Supplementary Table 9: Peak year of total greenhouse gas (GHG) emissions, GHG emissions in 2030 and 2050 (relative to 2015), phase-out year of GHG emissions (as in Figure 1), and negative emissions in 2100, per country and for 2 °C and 1.5 °C scenarios. Median [minimum; maximum].

	Peak year of GHG emissions (-)		GHG emissions in 2030, relative to 2015 (%)		GHG emissions in 2050, relative to 2015 (%)		Phase-out year of GHG emissions (-)		Negative emissions in 2100 ($Mt\ CO_2$ / year)	
	2 °C	1.5 °C	2 °C	1.5 °C	2 °C	1.5 °C	2 °C	1.5 °C	2 °C	1.5 °C
Brazil	2020 [2005-2045]	2015 [2005-2020]	-33 [-99; -10]	-50 [-114; -42]	-52 [-201; 41]	-72 [-213; -59]	2037 [2035-2040]	2040 [2030-2135]	481 [262-692]	621 [230-713]
Canada	2005 [2005-2010]	2005 [2005-2010]	-36 [-37; -19]	-43 [-78; -16]	-60 [-88; -42]	-78 [-110; -69]	2113 [2100-2126]	2045 [2045-2045]	440 [307-664]	402 358-436]
China	2020 [2020-2025]	2020 [2020-2020]	-28 [-55; 4]	-45 [-61; -23]	-66 [-81; -48]	-82 [-91; -66]	2100 [2080-2186]	2070 [2060-2090]	2662 [656-4062]	2937 [491-4720]
EU	2005 [2005-2010]	2005 [2005-2010]	-28 [-52; -20]	-41 [-72; -32]	-65 [-81; -38]	-81 [-100; -75]	2100 [2080-2116]	2070 [2060-2090]	1687 [889-2505]	1605 [889-3141]
India	2020 [2020-2050]	2020 [2020-2020]	-12 [-51; 27]	-25 [-54; 8]	-53 [-67; 25]	-68 [-94; -19]	2130 [2070-2183]	2090 [2060-2143]	917 [686-6029]	1047 [542-5256]
Indonesia	2020 [2010-2020]	2020 [2010-2020]	-36 [-46; -27]	-48 [-71; -28]	-66 [-83; -44]	-73 [-102; -68]	2090 [2070-2100]	2080 [2050-2114]	776 [363-989]	622 [229-1104]
Japan	2005 [2005-2020]	2005 [2005-2015]	-36 [-63; -19]	-47 [-55; -24]	-70 [-103; -58]	-85 [-116; -77]	2080 [2050-2090]	2065 [2045-2080]	530 [209-1081]	475 [344-876]
Russia	2015 [2015-2020]	2015 [2015-2020]	-28 [-45; -21]	-57 [-79; -21]	-63 [-85; -23]	-74 [-94; -63]	2085 [2080-2090]	2070 [2070-2117]	812 [413-1647]	934 [610-2422]
Turkey	2020 [2020-2020]	2020 [2020-2020]	-13 [-29; -5]	-28 [-41; -11]	-60 [-64; -28]	-82 [-93; -51]	2146 [2070-2155]	2070 [2060-2100]	240 [232-258]	188 [151-338]
USA	2005 [2005-2020]	2005 [2005-2020]	-35 [-61; -16]	-46 [-68; -34]	-72 [-84; -51]	-90 [-102; -81]	2080 [2060-2125]	2060 [2050-2070]	2960 [2187-3902]	2951 [2292-5070]
World	2020 [2020-2020]	2020 [2010-2020]	-25 [-47; -12]	-37 [-61; -25]	-65 [-72; -35]	-82 [-100; -63]	2090 [2080-2156]	2070 [2050-2070]	24316 [14214-31214]	22439 [15959-31914]

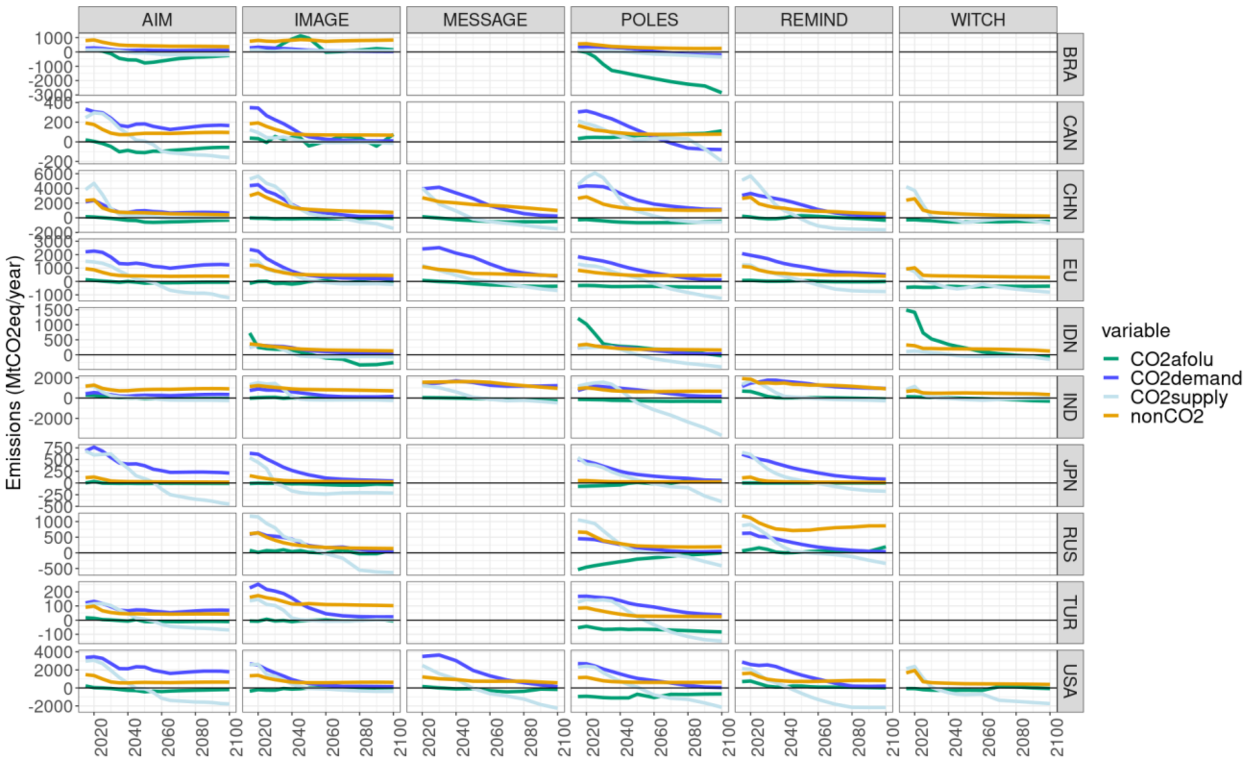

Supplementary Figure 6: Emission pathways per model and region. Emission pathways per model and region, aggregated to: AFOLU CO_2 emissions, energy demand CO_2 emissions, energy supply CO_2 emissions, and non-CO_2 emissions. 2 °C scenario only.

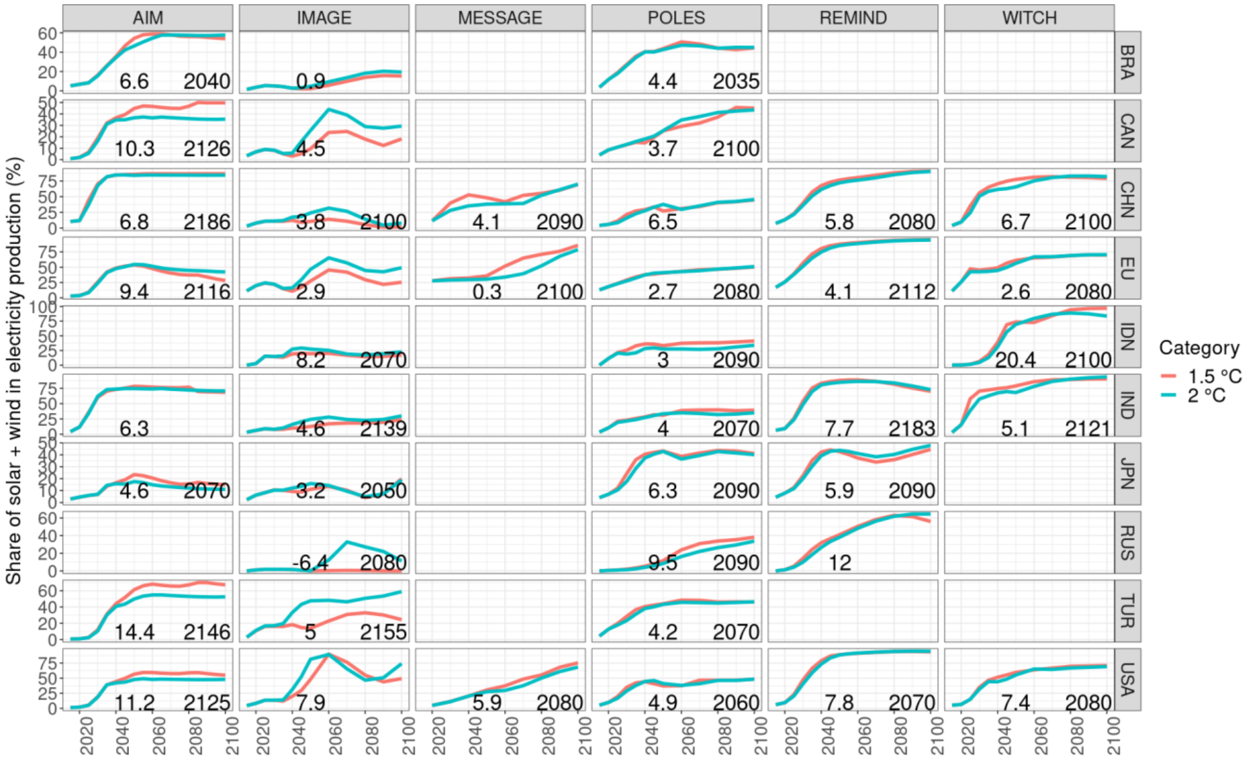

Supplementary Figure 7: Share of solar and wind power in total electricity production. Share of solar and wind power in total electricity production (%), per model and region, for 1.5 °C and 2 °C scenarios. Numbers show the rate of change (as compound annual growth rate, % per year over 2020-2050) for the 2 °C scenario, as well as the year total GHG emissions reach net zero in the 2 °C scenario.

Supplementary References

1 UNFCCC. *Greenhouse Gas Inventory Data - Detailed data by Party*, <http://di.unfccc.int/detailed_data_by_party> (2019).

2 UNFCCC. *GHG Profiles - Non-Annex I* <https://di.unfccc.int/ghg_profile_non_annex1> (2019).

3 Kuramochi, T. *et al.* Greenhouse gas mitigation scenarios for major emitting countries - Analysis of current climate policies and mitigation commitments: 2019 Update (NewClimate Institute, Cologne, Germany, 2019).

4 SEEG. *Total emissions*, <http://plataforma.seeg.eco.br/total_emission#> (2018).
